# Chronic Inflammatory Diseases and Cardiovascular Risk: Current Insights and Future Strategies for Optimal Management

**DOI:** 10.3390/ijms26073071

**Published:** 2025-03-27

**Authors:** Stefano Cacciatore, Silvia Andaloro, Marco Bernardi, Armando Oterino Manzanas, Luigi Spadafora, Stefano Figliozzi, Elad Asher, Jamal S. Rana, Fiona Ecarnot, Felice Gragnano, Paolo Calabrò, Antonio Gallo, Giuseppe Andò, Stephane Manzo-Silberman, Jeanine Roeters van Lennep, Matteo Tosato, Francesco Landi, Giuseppe Biondi-Zoccai, Emanuele Marzetti, Pierre Sabouret

**Affiliations:** 1 Department of Geriatrics, Orthopedics and Rheumatology, Università Cattolica del Sacro Cuore, Largo F. Vito 1, 00168 Rome, Italy; francesco.landi@unicatt.it; 2 Fondazione Policlinico Universitario “Agostino Gemelli” IRCCS, Largo A. Gemelli 8, 00168 Rome, Italy; matteo.tosato@policlinicogemelli.it; 3 Department of Translational Medicine and Surgery, Università Cattolica del Sacro Cuore, Largo F. Vito 1, 00168 Rome, Italy; silvia.andaloro01@icatt.it; 4 Department of Medical-Surgical Sciences and Biotechnologies, Sapienza University of Rome, Corso della Repubblica 79, 04100 Latina, Italy; marco.bernardi23@gmail.com (M.B.); luigispadafora167@gmail.com (L.S.); giuseppe.biondizoccai@uniroma1.it (G.B.-Z.); 5 Department of Cardiology, Hospital Universitario de Salamanca-IBSAL, Paseo de San Vicente, 58-182, 37007 Salamanca, Spain; aoterino@usal.es; 6 IRCCS Humanitas Research Hospital, Via Alessandro Manzoni, 56, Rozzano, 20089 Milano, Italy; stefano.figliozzi@humanitas.it; 7 Department of Biomedical Sciences, Humanitas University, Via Rita Levi Montalcini, 4, Pieve Emanuele, 20090 Milano, Italy; 8 Jesselson Integrated Heart Center, The Eisenberg R&D Authority, Shaare Zedek Medical Center, Faculty of Medicine, Hebrew University of Jerusalem, Shmuel (Hans) Beyth St. 12, Jerusalem 9103102, Israel; el.asher@gmail.com; 9 Division of Cardiology, Kaiser Permanente Northern California, 1 Kaiser Plaza, Oakland, CA 94612, USA; jamal.s.rana@kp.org; 10 Division of Research, Kaiser Permanente Northern California, 1 Kaiser Plaza, Oakland, CA 94612, USA; 11 Department of Cardiology, University Hospital, Boulevard Fleming, 25000 Besançon, France; fiona.ecarnot@univ-fcomte.fr; 12 SINERGIES Unit, University Marie & Louis Pasteur, 19 Rue Ambroise Paré, 25000 Besançon, France; 13 Department of Translational Medical Sciences, University of Campania “Luigi Vanvitelli”, Via Leonardo Bianchi, Ospedale Monaldi, 80131 Naples, Italy; felice.gragnano@unicampania.it (F.G.); paolo.calabro@unicampania.it (P.C.); 14 Division of Cardiology, A.O.R.N. “Sant’Anna e San Sebastiano”, Via Ferdinando Palasciano, 81100 Caserta, Italy; 15 INSERM UMR1166, IHU ICAN, Lipidology and Cardiovascular Prevention Unit, Department of Nutrition, Pitié-Salpêtrière Hospital, Sorbonne University, AP-HP, 47–83 Bd de l’Hôpital, 75013 Paris, France; antonio.gallo@aphp.fr; 16 Department of Clinical and Experimental Medicine, University of Messina, Azienda Ospedaliera Universitaria Policlinico “Gaetano Martino”, Via Consolare Valeria, 1, 98124 Messina, Italy; giuseppe.ando@unime.it; 17 ACTION Study Group, Inserm UMRS1166, Heart Institute, Pitié-Salpetriere Hospital, Sorbonne University, 47-83 Bd de l’Hôpital, 75013 Paris, France; stephane.manzosilberman@aphp.fr (S.M.-S.); cardiology.sabouret@gmail.com (P.S.); 18 Department of Internal Medicine, Cardiovascular Institute, Erasmus Medical Center, Dr. Molewaterplein 40, 3015 GD Rotterdam, The Netherlands; j.roetersvanlennep@erasmusmc.nl; 19 Maria Cecilia Hospital, GVM Care & Research, Via Corriera, 1, 48033 Cotignola, Italy

**Keywords:** atherosclerosis, risk assessment, cardiovascular diseases, chronic inflammation, inflammatory bowel diseases, rheumatoid arthritis, systemic lupus erythematosus, immunomodulation, anti-inflammatory agents, biomarkers

## Abstract

Chronic inflammation is a pivotal driver in the progression of atherosclerosis, significantly contributing to the burden of cardiovascular disease (CVD). Patients with chronic inflammatory diseases, such as inflammatory bowel diseases (IBDs) (e.g., ulcerative colitis and Crohn’s disease), rheumatological disorders, as well as individuals with auto-immune diseases (such as systemic lupus erythematosus), present a higher risk of major adverse cardiac events (MACEs). Despite their elevated CVD risk, these populations remain underrepresented in cardiovascular research, leading to a critical underestimation of their cardiovascular risk (CVR) in clinical practice. Furthermore, even recent CVR scores poorly predict the risk of events in these specific populations. This narrative review examines the physiopathological mechanisms linking chronic inflammation, immunomodulation, atherosclerosis, thrombosis and cardiovascular events. We review data from epidemiological studies and clinical trials to explore the potential cardiovascular benefits of anti-inflammatory and immunomodulatory therapies. Despite existing evidence, significant gaps in knowledge remain. Future research is mandatory, focusing on innovative strategies for risk stratification and optimization, including lipidomics, proteomics, advanced inflammatory markers, microbiota profiling, and cardiovascular imaging. Addressing these unmet needs will enhance understanding of cardiovascular risk in chronic inflammatory diseases, enabling tailored interventions and better outcomes.

## 1. Introduction

Chronic inflammatory diseases (CIDs), such as inflammatory bowel diseases (IBDs), rheumatoid arthritis (RA), systemic lupus erythematosus (SLE), and spondyloarthropathies, are characterized by persistent immune system activation, leading to systemic inflammation and multi-organ involvement. Increasing evidence highlights a strong link between chronic inflammation and cardiovascular disease (CVD), with epidemiological studies confirming an elevated risk of cardiovascular events in CID populations, independent of traditional risk factors such as hypertension, dyslipidemia, diabetes, and smoking [1,2,3,4,5,6]. Patients with CIDs experience a heightened burden of cardiovascular morbidity and mortality, driven by systemic inflammation that fosters a pro-atherogenic state [7]. Chronic immune activation contributes to endothelial dysfunction [7], oxidative stress [8,9], and vascular remodeling [10], which collectively accelerate the development of atherosclerosis and increase the likelihood of major adverse cardiovascular events (MACEs), including myocardial infarction, stroke, and heart failure [4,11].

Recognizing the cardiovascular implications of chronic inflammation has sparked interest in novel therapeutic strategies aimed at mitigating inflammation-driven CVD risk. These approaches include the repurposing of anti-inflammatory agents such as tumor necrosis factor (TNF) inhibitors, interleukin (IL)-6 blockers, and Janus kinase (JAK) inhibitors, alongside emerging biologics with immunomodulatory effects. However, concerns persist regarding the cardiovascular safety of certain immunosuppressive therapies, necessitating further research into their long-term risk–benefit profile [12]. The objective of this review is to provide a comprehensive description of the relationship between CIDs and CVD, emphasizing the underlying molecular and immunological mechanisms that contribute to elevated cardiovascular risk in affected individuals. Additionally, we examine current and emerging strategies for optimal cardiovascular risk management in patients with CIDs, encompassing pharmacological interventions that target inflammatory pathways, lifestyle modifications, and integrative approaches to risk stratification.

## 2. Overview of Chronic Inflammatory Diseases

CIDs encompass a wide range of disorders characterized by persistent inflammation and systemic involvement. However, the present review focuses primarily on IBDs, RA, and SLE, which are among the most extensively studied. We excluded systemic and localized vasculitides, as these primarily affect blood vessels and the heart directly through vascular inflammation, thrombosis, and endothelial dysfunction, leading to distinct CVD risk profiles [13].

### 2.1. Inflammatory Bowel Diseases

IBD, which includes ulcerative colitis and Crohn’s disease, affects approximately 1% of the global population, with an increasing incidence worldwide. Symptoms include abdominal pain, chronic diarrhea, weight loss, and hematochezia, with alternating periods of remission and exacerbation [14]. In older adults, IBD more frequently involves the distal intestine and is associated with higher rates of complications, such as ischemic colitis, infectious colitis, colorectal cancer, and perforation [15].

The pathogenesis of IBD is complex and involves genetic predisposition, environmental factors, and dysregulation of both innate and adaptive immunity [16]. Ulcerative colitis is restricted to the colon, affecting only the mucosal layer, and typically begins in the rectum, extending proximally. In contrast, Crohn’s disease can affect the entire gastrointestinal tract, from the mouth to the anus, with transmural inflammation, skip lesions, and granuloma formation [14].

Beyond the gastrointestinal tract, chronic systemic inflammation in IBD significantly impacts cardiovascular health. Systemic inflammation driven by gut dysbiosis and altered immune signaling contributes to endothelial dysfunction, arterial stiffness, and fosters a prothrombotic state [17,18]. Studies have demonstrated that patients with IBD exhibit an increased risk of myocardial infarction, heart failure, and stroke, independent of traditional cardiovascular risk factors [19,20]. Moreover, IBD patients, particularly those with active disease, have a heightened risk of venous thromboembolism, with an up to threefold increased likelihood of deep vein thrombosis and pulmonary embolism compared to the general population [21]. Recent clinical trials and observational studies have highlighted the impact of immunosuppressive therapies on cardiovascular risk in IBD. While biologic agents such as anti-TNF inhibitors (e.g., infliximab, adalimumab) have been associated with a reduction in cardiovascular events [22], JAK inhibitors may carry a higher thromboembolic risk [23]. Corticosteroids, commonly used to control IBD flares, are associated with worsening cardiometabolic profiles, including increased blood pressure, insulin resistance, and dyslipidemia [22]. Understanding the interplay between systemic inflammation, gut microbiota, and cardiovascular risk in IBD is crucial for developing targeted preventive strategies.

Management of IBD focuses on reducing inflammation, achieving remission, and preventing relapses. Treatment options include 5-aminosalicylic acid drugs, corticosteroids, immunomodulators (e.g., azathioprine, methotrexate), and biologic agents (e.g., infliximab, vedolizumab) [24,25]. Surgery is reserved for refractory cases, complications, or colorectal cancer risk [26,27].

### 2.2. Rheumatoid Arthritis

RA is a systemic autoimmune disorder affecting approximately 1% of the population, with a higher prevalence in women (3:1 female-to-male ratio). The disease primarily targets synovial joints, leading to chronic synovitis, progressive joint destruction, and functional disability. Autoantibodies, including rheumatoid factor and anti-citrullinated protein antibodies, drive immune-mediated joint inflammation and systemic complications such as cardiovascular disease, interstitial lung disease, and osteoporosis [28].

RA management aims to control inflammation, prevent joint damage, and maintain function through a combination of pharmacologic and non-pharmacologic approaches. Methotrexate is the first-line disease-modifying antirheumatic drug (DMARD) [29], while biologic and targeted synthetic DMARDs (e.g., TNF inhibitors, IL-6 inhibitors, JAK inhibitors) are used in cases of inadequate response or decreased effectiveness after initial treatment success [30]. Lifestyle interventions, including smoking cessation, exercise, and dietary modifications, play a critical role in disease management and CVD risk reduction [31].

### 2.3. Systemic Lupus Erythematosus

SLE is a multisystem autoimmune disease that predominantly affects women of childbearing age [32]. It presents with diverse clinical manifestations, including malar rash, arthritis, nephritis, hematologic abnormalities, and serositis. Patients with SLE have a markedly increased risk of cardiovascular disease, with rates of myocardial infarction up to 50 times higher in young women with SLE compared to age-matched controls [33]. Chronic systemic inflammation, immune complex deposition, and dysregulated interferon signaling contribute to endothelial dysfunction, accelerated atherosclerosis, and arterial thrombosis [34]. Additionally, SLE is associated with a high prevalence of metabolic syndrome, dyslipidemia, and insulin resistance, further exacerbating cardiovascular risk [35]. Antiphospholipid syndrome, which frequently coexists with SLE, further compounds cardiovascular risk by promoting a hypercoagulable state, increasing the risk of arterial and venous thrombosis [36]. Therapeutic strategies for cardiovascular prevention in SLE are evolving [32,36]. Hydroxychloroquine, a cornerstone of lupus treatment, has demonstrated cardioprotective effects [37]. Conversely, prolonged corticosteroid therapy is associated with an increased incidence of hypertension, diabetes, and atherosclerosis, necessitating careful risk–benefit assessment [38]. Emerging biologic agents, such as anifrolumab (an interferon receptor antagonist) [39] and belimumab (a B-cell inhibitor) [40], may offer cardiovascular benefits by reducing systemic inflammation, though long-term cardiovascular outcomes require further investigation.

## 3. Pathophysiology of Inflammation and Cardiovascular Disease

Atherosclerosis is a complex and multifactorial disease that extends beyond the passive accumulation of cholesterol-bound lipoproteins. Instead, it is driven by chronic inflammation, oxidative stress, immune dysregulation, and endothelial dysfunction, all of which contribute to plaque formation and MACE.

Endothelial dysfunction is an early and central event in atherogenesis, promoting increased vascular permeability, leukocyte adhesion, and oxidative stress. The loss of the protective endothelial glycocalyx allows for the infiltration of inflammatory cells and oxidized lipoproteins into the arterial intima, fostering the development of foam cells and atheromatous plaques. Persistent immune activation, characterized by elevated levels of tumor necrosis factor-alpha (TNF-α), IL-1β, and IL-6, exacerbates vascular remodeling and plaque instability, thereby increasing the risk of both MACE and thrombotic events [7]. Oxidative stress has been demonstrated to enhance lipid oxidation and foam cell formation, thereby contributing to plaque instability [8,9]. Immune dysregulation involving macrophages, T-helper cells, and regulatory T cells has been shown to perpetuate a pro-thrombotic state, leading to vascular remodeling and heightened risk of MACE [10]. The dysfunction of endothelial progenitor cells in these patients further exacerbates vascular repair deficits, accelerating atherogenesis [41]. The cumulative impact of these mechanisms results in an absolute excess cardiovascular risk among patients with CIDs, positioning chronic inflammation as a key cardiovascular risk enhancer.

### Key Inflammatory Markers

Atherosclerotic plaques are heavily infiltrated with inflammatory cells, including macrophages, T lymphocytes, and dendritic cells. Plaque rupture can trigger a systemic inflammatory response, detectable through various biomarkers.

C-reactive protein (CRP) is an acute-phase reactant produced in response to IL-6 stimulation. Research indicates CRP as a key predictor of MACE [42]. Elevated CRP levels are strongly correlated with increased mortality in coronary artery disease [43]. Fibrinogen is a coagulation factor that enhances platelet aggregation and thrombosis, with higher plasma levels linked to worse outcomes in ischemic stroke and myocardial infarction [44,45]. IL-6 and IL-1β are cytokines that drive vascular inflammation and plaque instability, with elevated levels associated with increased cardiovascular mortality [46,47]. Interestingly, their inhibition using monoclonal antibodies has been explored for secondary cardiovascular protection in high-risk patients (e.g., after myocardial infarction). The CANTOS trial investigated IL-1β inhibition using canakinumab in patients with a prior myocardial infarction and persistently elevated high-sensitivity C-reactive protein (hsCRP) levels equal to or greater than 2 mg/L. The study found that canakinumab significantly reduced MACE, with the greatest benefit observed in patients who achieved hsCRP levels below 2 mg/L following the initial dose. In patients with chronic kidney disease, canakinumab demonstrated an 18% reduction in the risk of MACE, with a more pronounced effect observed in individuals who exhibited a robust anti-inflammatory response [48]. The RESCUE trial demonstrated that ziltivekimab, an IL-6 inhibitor, significantly reduced hsCRP levels in a dose-dependent manner (up to 92% reduction) in patients with chronic kidney disease and high cardiovascular risk. Additionally, it was observed to decrease other inflammatory and thrombotic biomarkers without affecting lipid ratios or causing serious adverse events [49].

Interferon-gamma (IFN-γ) is a pro-inflammatory cytokine primarily produced by T cells and macrophages that plays an important role in the progression of atherosclerosis. IFN-γ promotes immune cell recruitment, enhances oxidative stress, increases foam cell formation, and destabilizes plaques by stimulating smooth muscle cell apoptosis and matrix metalloproteinase production. It is also a critical activator of the JAK/signal transducer and activator of transcription (STAT) signaling pathway, which drives the inflammatory cascade in atherosclerosis [50]. Elevated IFN-γ levels have been associated with increased plaque vulnerability, endothelial dysfunction, and adverse cardiovascular outcomes [51]. Given its role in cardiovascular disease, IFN-γ and its signaling pathways have been explored as possible therapeutic targets. Potential treatments include monoclonal antibodies like AMG811, which selectively neutralizes IFN-γ, and JAK inhibitors like baricitinib and tofacitinib, which suppress downstream IFN-γ signaling. However, substantial benefits on CVD risk and MACE have not been demonstrated for any of these treatments [50]. Additionally, molecules such as resveratrol and adenosine have demonstrated the potential to modulate IFN-γ activity by preventing STAT1 phosphorylation, though clinical evidence remains inconclusive [50]. Nonetheless, long-term IFN-γ inhibition poses risks due to its essential role in host immunity, necessitating further research into balancing its cardiovascular effects with immune protection [50].

Emerging evidence suggests that endothelial glycocalyx degradation products may serve as a marker of vascular dysfunction, warranting further research into glycocalyx-restoring therapies [52]. Finally, loss of endothelial integrity leads to increased expression of pro-thrombotic molecules (P-selectin, E-selectin, tissue factor) that enhance clot formation and inflammation [53,54].

Targeting inflammatory pathways offers a promising avenue for cardiovascular risk reduction. Colchicine, methotrexate, and leukotriene inhibitors have demonstrated potential benefit by attenuating vascular inflammation and reducing cardiovascular events [55,56,57].

## 4. Clinical Trials on Cardiovascular Risk and Chronic Inflammatory Diseases

Over the past two decades, numerous clinical trials have investigated pharmacological and non-pharmacological interventions aimed at mitigating CVD risk in patients with CIDs. The effects of DMARDs, biologic therapies, lipid-lowering agents, alternative treatments, and structured cardiovascular risk management strategies are summarized in Table 1.

### 4.1. Biologic and Targeted Synthetic Disease-Modifying Therapies: Cardiovascular Benefits and Risks

RA has been extensively studied regarding the cardiovascular effects of biologic and targeted synthetic DMARDs. Several trials highlight the potential benefits and risks associated with these therapies. Methotrexate, a cornerstone DMARD in RA, has been shown to improve vascular function and reduce carotid intima–media thickness, suggesting a protective role against atherosclerosis [71,74]. Infliximab, a TNF inhibitor, has demonstrated the ability to slow atherosclerosis progression in RA patients [65]. Additionally, studies indicate that methotrexate, either alone or combined with TNF inhibitors, may reduce glycocalyx degradation markers, such as syndecan-1, supporting its vascular protective role [62]. However, concerns remain regarding the cardiovascular safety of JAK inhibitors. While baricitinib and tofacitinib have shown efficacy in controlling RA disease activity, trials indicate an increased risk of MACE, particularly in older patients and smokers [63,73]. Upadacitinib, another JAK inhibitor, demonstrated superior efficacy in achieving remission compared to abatacept but was associated with a higher incidence of MACE [75,76]. Trials comparing abatacept (targeting the human cytotoxic T-lymphocyte-associated antigen 4) and adalimumab (TNF inhibitor) in RA patients demonstrated that both therapies improved high-density lipoprotein (HDL) functionality; however, adalimumab had a stronger effect on increasing paraoxonase-1 activity and reducing HDL-associated inflammatory markers [60]. These findings highlight that certain biologics may have more favorable lipid-modulating properties, which could impact long-term cardiovascular outcomes in RA patients.

In IBD, the long-term extension of the UNIFI trial showed that subcutaneous ustekinumab maintained clinical remission and endoscopic improvement for four years, with no MACE reported, though MACE was not a primary endpoint [86]. Similarly, the UNITI extension study on Crohn’s disease demonstrated durable remission up to 92 weeks without an increase in serious adverse events or cardiovascular complications [88]. A trial on ozanimod, a sphingosine-1-phosphate receptor modulator for ulcerative colitis, reported no significant cardiovascular safety concerns [87].

In SLE, studies evaluating anifrolumab, an interferon receptor blocker, showed promising reductions in inflammatory markers and cholesterol efflux capacity, which may contribute to cardiovascular protection [39]. Long-term data from Wallace et al. [40] showed that belimumab maintained disease control for up to 13 years, with a low incidence of cardiovascular deaths, although cardiovascular outcomes were not a primary focus.

### 4.2. Targeting Inflammatory Pathways for Cardiovascular Risk Reduction

Recent clinical trials have underscored the potential of colchicine as an anti-inflammatory therapy for CVD. The Colchicine Cardiovascular Outcomes Trial (COLCOT) [89] demonstrated that low-dose colchicine (0.5 mg daily) significantly reduced major adverse cardiovascular events (MACEs), including stroke and hospitalization for angina requiring revascularization, in patients with a recent myocardial infarction. Similarly, the LoDoCo2 trial [90] found that colchicine lowered cardiovascular event rates in patients with stable coronary artery disease, supporting its role in secondary prevention. However, findings from the CHANCE-3 trial [91] suggested that while colchicine exhibits anti-inflammatory and atheroprotective effects, its benefits in preventing ischemic stroke and cardiovascular events remain inconsistent. Likewise, the CLEAR trial [92] indicated that colchicine may have limited efficacy in the acute phase post-myocardial infarction, suggesting that its cardiovascular benefits are influenced by timing and patient selection. These findings highlight the need for further studies to identify optimal patient populations and refine the role of colchicine in cardiovascular risk reduction.

IL-1 and IL-6 inhibitors have been explored for their cardiovascular benefits in inflammatory diseases. In RA, anakinra (IL-1 inhibitor) improved myocardial function, while tocilizumab (IL-6 inhibitor) enhanced vascular function [69,70]. However, clinical trials assessing IL-6 inhibition have reported mixed results [78]. While tocilizumab effectively reduces systemic inflammation, it has been associated with increased lipid levels, raising questions about its net cardiovascular benefit [64].

Similarly, targeted therapies in SLE, such as cenerimod (sphingosine-1-phosphate modulator) and tofacitinib (JAK inhibitor), have demonstrated improvements in lipid function and endothelial health, but their long-term cardiovascular implications remain uncertain [80,83].

### 4.3. Lipid-Lowering Strategies: The Role of Statins in Inflammatory Diseases

Statins play a crucial role in mitigating cardiovascular risk in CID populations. Their lipid-lowering and anti-inflammatory properties make them particularly beneficial for patients with chronic inflammation.

RA studies have demonstrated the efficacy of rosuvastatin in improving arterial stiffness and subendocardial viability ratio, though its impact on carotid intima–media thickness remains inconclusive [77]. Long-term studies confirm statin benefits in reducing arterial stiffness and blood pressure in inflammatory joint diseases [67,68]. Similarly, atorvastatin has been shown to improve lipid profiles and inflammation in RA patients, with recent trials reporting a significant reduction in cardiovascular event risk [61,72].

In SLE, a randomized controlled trial by Plazak et al. [85] demonstrated that atorvastatin halted the progression of coronary artery calcifications and improved myocardial perfusion using imaging endpoints. Fatemi et al. [82] found that atorvastatin improved lipid profile and inflammatory markers in SLE, but the short follow-up precluded assessment of cardiovascular event reduction. Similarly, Mok et al. [84] reported that rosuvastatin lowered homocysteine and endothelial activation markers in SLE patients with subclinical atherosclerosis but had limited impact on carotid intima–media thickness progression. While research on statins in IBD and SLE is limited, given their overall cardiovascular protective effects, they may be considered an essential component of CVD risk management in CID populations [81].

### 4.4. Alternative and Complementary Approaches to Cardiovascular Risk Reduction

In addition to pharmacological interventions, alternative approaches have been explored for reducing cardiovascular risk in CIDs. Puerarin, a bioactive compound from traditional Chinese medicine, has been investigated for its potential benefits in RA patients, showing reductions in carotid intima–media thickness and insulin resistance [79]. However, larger placebo-controlled trials are required before it can be recommended for routine use.

Exercise-based interventions, including high-intensity interval training (HIIT), have been studied in inflammatory rheumatic diseases. Trials suggest that supervised and smartphone-assisted HIIT programs can improve VO2max and overall cardiovascular health in these populations, highlighting the role of structured physical activity in cardiovascular risk management [66].

### 4.5. Treat-to-Target Strategies for Cardiovascular Risk Management

Structured cardiovascular risk management programs have shown promise in improving cardiovascular outcomes in CID patients. Treat-to-target (T2T) strategies, which involve aggressive control of inflammation and metabolic risk factors, have been tested in RA. Trials indicate that carotid intima–media thickness progression is significantly lower in RA patients following T2T approaches, particularly in those without metabolic syndrome [58,59].

### 4.6. Lessons Learned and Perspectives

The cumulative evidence highlights the complex interplay between chronic inflammation and cardiovascular risk, emphasizing the need for integrated treatment strategies. Aggressive lipid lowering with statins, careful selection of biologic and targeted synthetic DMARDs, and structured cardiovascular risk management programs can significantly improve long-term cardiovascular outcomes in CID patients. While biologics, particularly TNF inhibitors and IL-6 blockers, show potential cardiovascular benefits, concerns persist about the cardiovascular safety of JAK inhibitors.

It is worth noting that dedicated clinical trials evaluating cardiovascular risk reduction strategies in patients with SLE and IBD are lacking. Most of the current evidence regarding cardiovascular risk in these populations stems from observational studies rather than randomized controlled trials, which limits the strength of the recommendations that can be made. Moreover, clinical trials in CIDs face several limitations that constrain our understanding of cardiovascular risk reduction strategies. Most are designed primarily to assess disease control and safety, with cardiovascular outcomes included only as secondary or exploratory endpoints. As a result, these trials are often underpowered to detect MACE, and follow-up durations are typically too short to capture long-term vascular effects. Patient populations are frequently unrepresentative of real-world clinical practice, with older adults, individuals with pre-existing cardiovascular disease, and those with multiple comorbidities commonly excluded. This limits the applicability of findings to high-risk groups. Many studies rely on surrogate markers, such as CRP or carotid intima–media thickness, which may not reliably predict clinical benefit. The limited use of advanced cardiovascular imaging and longitudinal biomarker monitoring reduces our ability to detect subclinical disease progression and therapeutic impact.

Future research should prioritize the refinement of cardiovascular risk stratification tools, the evaluation of novel preventive and therapeutic strategies, and the long-term assessment of immunomodulatory therapies in patients with chronic inflammatory diseases. Integrating cardiovascular risk management into routine care, particularly within rheumatology, gastroenterology, and dermatology, will be essential to mitigate excess cardiovascular morbidity and mortality. A multidisciplinary, proactive approach has the potential to significantly improve both cardiovascular and overall outcomes in this high-risk population.

## 5. Management Strategies

The optimal management of CVD risk in patients with CIDs requires a multicomponent approach that integrates regular screening, patient empowerment, risk stratification beyond traditional models, timely lipid-lowering therapy, and comprehensive lifestyle modifications (Table 2).

### 5.1. Regular Screening and Cardiovascular Risk Stratification

Traditional cardiovascular risk scores, such as SCORE2 for 10-year risk of MACE or Life-CVD2 for lifetime risk, estimate the risk of major cardiovascular events but do not fully account for the pro-inflammatory burden of CIDs. As a result, cardiovascular risk in patients with CIDs may be underestimated, leading to delayed intervention [93]. To improve risk prediction and early detection, a more refined screening strategy should be implemented, particularly in CID patients who may develop subclinical atherosclerosis despite normal low-density lipoprotein (LDL) cholesterol levels. Among available risk models, the Reynolds Risk Score [94] and QRISK [95] have been shown to be more adapted to risk estimation in RA and SLE patients, as they integrate inflammatory markers and chronic disease burden.

Proactive cardiovascular risk assessment should be integrated into routine care, particularly in patients with long-standing disease or frequent flares, including comprehensive evaluation that integrates CVD risk biomarkers, including lipoprotein(a) [96], inflammatory biomarkers (e.g., CRP, IL-6, fibrinogen), chronic inflammatory burden, and disease activity scores. Non-invasive imaging techniques, including carotid intima–media thickness, coronary artery calcium (CAC) scoring, and echocardiography, may help to assess early vascular damage [97]. Cardiovascular risk management should account for disease activity and corticosteroid exposure [17,98]. In SLE, antiphospholipid antibody status should be assessed, with aggressive thromboembolism prevention strategies for high-risk individuals [36]. Frequent cardiovascular assessments should be performed in high-risk patients, especially older adults, those with long-standing disease, or those receiving therapies with known cardiovascular risks (e.g., JAK inhibitors) [99,100].

### 5.2. The Role of Cardiovascular Imaging in Chronic Inflammatory Diseases

Cardiovascular imaging plays a crucial role in the early detection and risk stratification of atherosclerosis in patients with CIDs [97].

Traditional imaging modalities remain foundational in clinical practice and provide substantial value in cardiovascular risk assessment. Carotid ultrasound is a non-invasive, widely available, and cost-effective method that enables the detection of carotid plaques and arterial thickening, which correlate with systemic atherosclerosis and cardiovascular events. Patients with CIDs frequently exhibit elevated levels of subclinical, early-stage atherosclerosis, which can be detected using carotid ultrasound [101,102]. CAC scoring, obtained via non-contrast computed tomography (CT), is one of the most validated imaging tools for cardiovascular risk stratification in asymptomatic individuals, including those with CIDs. Emerging evidence suggests that incorporating CAC scoring into cardiovascular risk assessment provides incremental predictive value beyond traditional risk factors and can unmask high-risk profiles that may be underestimated by conventional scoring systems [103,104]. Moreover, it significantly improves the prediction of MACE in CID populations [103,105]. Coronary CT angiography is another well-established technique that offers comprehensive visualization of coronary anatomy, plaque burden, and stenosis severity, aiding in the identification of subclinical coronary artery disease [106,107].

Among emerging imaging modalities, photon-counting computed tomography (PCCT) represents a significant advancement over conventional CT imaging, offering improved spatial resolution, superior tissue characterization, and enhanced signal-to-noise ratio [108]. A key advantage of PCCT in cardiovascular imaging is its ability to detect low-attenuation plaques, which are highly predictive of future adverse cardiovascular events [109]. These plaques, characterized by a necrotic core and lipid-rich composition, are more prone to rupture, leading to acute coronary syndromes. PCCT enables the visualization of these vulnerable plaques with greater accuracy than conventional CT, providing clinicians with an early warning sign of atherosclerotic disease progression [109]. Additionally, PCCT enhances the assessment of vascular inflammation by offering higher contrast resolution, enabling better detection of arterial wall thickening, microcalcifications, and early-stage fibrosis [110]. Beyond plaque characterization, PCCT improves the evaluation of CAC, a well-established marker of subclinical atherosclerosis [111]. This refined analysis is particularly relevant for CID patients, as they often develop atherosclerosis at an accelerated rate [112].

### 5.3. Patient Empowerment in Cardiovascular Risk Management

Empowering patients with knowledge and self-monitoring tools can significantly improve cardiovascular outcomes [113,114]. Regular home blood pressure monitoring is essential, as hypertension is a common but modifiable risk factor for CVD. In addition, awareness campaigns highlighting the link between chronic inflammation and CVD can educate patients about their increased cardiovascular risk, even when traditional risk factors are well controlled. Promoting lifestyle accountability also empowers patients to actively track their diet, exercise and adhere to their medication regimen through mobile health applications or regular check-ins with healthcare providers, fostering long-term engagement in their cardiovascular health.

### 5.4. Accounting for CIDs as Risk Modifiers in Cardiovascular Risk Estimation

A major limitation of current cardiovascular risk models is their failure to account for CIDs as independent risk modifiers [93]. The burden of CIDs should be explicitly incorporated into 10-year and lifetime cardiovascular risk assessment models to ensure that appropriate preventive measures are implemented. Personalized risk assessment algorithms should incorporate disease duration, severity, and inflammatory activity into CVD risk calculation. Stronger preventive efforts, including earlier initiation of statins and tighter blood pressure control, should be considered for CID patients even if traditional risk scores suggest low or moderate risk.

### 5.5. Lipid-Lowering Strategies: Beyond LDL Reduction

Statin therapy remains a cornerstone of cardiovascular prevention, particularly in patients with CVD, where its anti-inflammatory effects provide additional vascular benefits. In addition to lowering LDL cholesterol, statins modulate inflammatory pathways, improving endothelial function and reducing pro-inflammatory cytokine production [115,116]. Early, appropriate, and long-term lipid-lowering therapy should be prioritized even in patients without overt dyslipidemia because of their underlying inflammatory risk. If LDL targets are not achieved, non-statin lipid-lowering agents (e.g., PCSK9 inhibitors, ezetimibe, bempedoic acid) may be added based on individual risk profiles. Statins should be an integral part of CID management, particularly in patients on JAK inhibitors or those with longer disease duration, because of their ability to reduce inflammation-related cardiovascular risk [117].

### 5.6. Lifestyle Modifications: The Foundation of Cardiovascular Prevention

Lifestyle modification remains the most effective and cost-effective strategy for long-term cardiovascular health in CID patients [118]. Smoking cessation is critical, as tobacco use increases inflammation and vascular damage [118]. Adherence to an anti-inflammatory diet, particularly the Mediterranean diet, which is rich in polyphenols, omega-3 fatty acids, and fiber, provides cardioprotective and anti-inflammatory benefit [119,120]. However, patients with Crohn’s disease and ulcerative colitis, particularly those with active disease or intestinal strictures, may require dietary modifications to reduce fiber intake while maintaining nutrient density and preventing malnutrition [121]. Finally, management of weight and body fat is essential, as obesity exacerbates inflammation and contributes to metabolic dysregulation, further increasing the risk of cardiovascular disease [122,123].

### 5.7. The Role of Physical Activity Across All Ages

Physical activity is fundamental to cardiovascular health, symptom management, and overall well-being in patients with CIDs [124]; however, exercise prescription should be tailored to age, disease severity, and functional capacity. In young adults, resistance training should be emphasized because it improves vascular function, reduces inflammatory markers, and maintains musculoskeletal health. In older adults, progressive resistance training should be integrated with balance and coordination exercises, which help reduce the risk of frailty, osteoporosis, and falls while improving cardiovascular markers [125]. HIIT may be beneficial for some patients, particularly those with inflammatory arthritis, as it has been shown to improve VO2 max and endothelial function [66]. Regardless of disease status, patients should be encouraged to incorporate daily exercise, even in the presence of fatigue or mild joint pain, as exercise has potent anti-inflammatory effects beyond its direct cardiovascular benefits [124].

### 5.8. Vaccination and Cardiovascular Risk Reduction in Chronic Inflammatory Diseases

Vaccination is a key strategy in reducing inflammation and preventing MACE in patients with CIDs. Respiratory infections such as influenza and pneumococcal pneumonia are linked to heightened systemic inflammation, endothelial dysfunction, and increased CVD risk, including acute coronary syndromes and stroke [126]. Patients with RA, SLE, IBDs, and other CIDs face a higher risk of severe infections due to immune dysregulation and immunosuppressive therapies, which can exacerbate inflammation, trigger disease flares, and elevate CVD risk. Following international guidelines, CID patients should receive influenza, pneumococcal (PCV13, PPSV23), tetanus toxoid, hepatitis A and B, HPV, and herpes zoster vaccines as part of routine preventive care [127,128]. Live vaccines (e.g., measles, mumps and rubella vaccination) should generally be avoided in immunosuppressed individuals but may be considered prior to immunosuppressive therapy initiation. Screening for hepatitis B, varicella-zoster virus, and HPV is recommended before starting immunosuppressive treatment, and whenever possible, vaccinations should be administered in advance to optimize immune response [127,128]. To improve vaccine coverage, healthcare providers should assess vaccination status annually, proactively recommend immunization, and address concerns about safety and efficacy, particularly in immunosuppressed patients [129]. Increasing awareness through patient education and provider engagement is crucial [130]. The use of electronic health records for vaccination reminders shows promise in improving adherence, though further research is needed to assess its full potential [131]. Future studies may refine vaccination strategies by improving efficacy data, optimizing booster schedules, and tailoring recommendations based on disease activity and immunosuppressive treatments.

### 5.9. Interdisciplinary Collaboration for Comprehensive Cardiovascular Risk Management in Chronic Inflammatory Diseases

Effective management requires a collaborative, multidisciplinary approach involving rheumatologists, gastroenterologists, cardiologists, internal medicine specialists, surgeons, nutritionists, and exercise specialists [132]. Rheumatologists and gastroenterologists should work closely with cardiologists to develop personalized cardiovascular risk reduction strategies. Internal medicine specialists help coordinate care for metabolic comorbidities such as hypertension and diabetes. Dietitians provide tailored nutritional guidance to optimize metabolic health and control inflammation, while exercise specialists facilitate structured physical activity programs aimed at improving cardiovascular fitness without exacerbating disease activity. Nurses play a critical role in patient education, adherence and ongoing monitoring to ensure early detection of complications and reinforce lifestyle changes. This integrated care model improves patient outcomes and ensures that cardiovascular risk reduction is seamlessly integrated into CID management [133,134].

## 6. Gaps in Knowledge, Emerging Research, and Future Directions

Despite significant progress in understanding cardiovascular risk in CID populations, several important gaps remain (Table 3). The ultimate goal of future research efforts should be to facilitate precision medicine, enabling individualized treatment strategies based on patient-specific molecular, genetic, and inflammatory profiles.

### 6.1. Improving Cardiovascular Risk Prediction in Chronic Inflammatory Diseases

Current cardiovascular risk assessment tools do not fully capture the impact of chronic inflammation on atherosclerosis and vascular dysfunction. The lack of large, longitudinal studies focusing on CID-related cardiovascular risk hinders accurate risk assessment and highlights the need for future research to address key gaps [93]. Refining risk prediction tools requires incorporating inflammatory markers, disease activity scores, and emerging multi-omics data. The latter include microbiome profiling to assess gut–immune interactions in CVD [18], metabolomics and lipidomics, to identify biochemical pathways linking chronic inflammation to atherogenesis [135], proteomics and transcriptomics to explore systemic inflammatory pathways and develop new biomarkers for cardiovascular risk [136].

In addition to traditional risk scores, polygenic risk scores (PRSs) have emerged as a promising tool for refining cardiovascular risk stratification. PRS aggregates the cumulative effect of multiple genetic variants associated with cardiovascular disease to estimate an individual’s inherited susceptibility to atherosclerosis, myocardial infarction, and other cardiovascular events. Unlike conventional risk models that primarily rely on clinical and biochemical parameters, PRS can help identify individuals at high cardiovascular risk even before clinical manifestations appear [137]. Further research may evaluate whether incorporating PRS into cardiovascular risk prediction models significantly improves risk stratification in populations with CIDs. In that way, PRS could serve as an early warning tool to prioritize closer cardiovascular surveillance and aggressive cardiovascular prevention strategies in CID patients, particularly those who do not exhibit traditional risk factors such as hyperlipidemia or hypertension. Moreover, PRS may offer insights into the differential cardiovascular risk across various CIDs subtypes.

Since autoimmune diseases disproportionately affect women, future studies should also explore sex-specific cardiovascular risk factors to develop tailored prevention strategies that account for differences in inflammatory burden, hormonal influences, and disease phenotype [138].

### 6.2. Role of Chronic Inflammation in Primary vs. Secondary Prevention

While chronic inflammation plays a well-established role in secondary cardiovascular prevention, its influence in primary prevention remains less understood [139]. A major research priority is to determine whether inflammation drives the first cardiovascular event with the same intensity as it does recurrent events, which would refine preventive treatment thresholds in CID populations. Targeted anti-inflammatory therapies, including colchicine, TNF inhibitors, IL-6 inhibitors, and IL-1 blockers, have demonstrated cardiovascular protective effects in select populations [139], but their long-term safety and efficacy in CID patients require further investigation. Future trials should compare the benefits of early intervention with immunomodulatory therapies versus conventional lipid-lowering and antihypertensive strategies to establish optimal management guidelines. Additionally, the role of gut microbiota in systemic inflammation and cardiovascular disease remains an area of active research. Dysbiosis, commonly observed in IBD and other inflammatory diseases, may contribute to atherogenesis through alterations in lipid metabolism, endothelial dysfunction, and immune activation [18]. Future studies should investigate the potential of gut-targeted interventions, such as probiotics and fecal microbiota transplantation, in reducing cardiovascular risk in CID populations. Moreover, the identification of novel, non-invasive biomarkers, such as oncostatin M, an IL-6 family cytokine, may improve risk stratification by more accurately predicting disease activity and cardiovascular risk, ultimately aiding in the development of targeted prevention strategies [140,141].

### 6.3. The Future of Cardiovascular Imaging in Chronic Inflammatory Diseases

The incorporation of advanced cardiovascular imaging techniques is critical for early atherosclerosis detection and personalized risk stratification in CID patients. PCCT and artificial intelligence (AI)-enhanced imaging allow for a more detailed characterization of coronary artery disease [142]. Future research should focus on validating PCCT and CAC scoring in CID-specific cardiovascular risk models, developing imaging-based inflammatory risk markers to assess disease activity and cardiovascular risk in real time, as well as exploring the cost-effectiveness and accessibility of advanced imaging for routine cardiovascular risk assessment in CID patients.

### 6.4. Integrating AI and Big Data into Clinical Practice

The integration of AI and machine learning algorithms into cardiovascular risk assessment holds great promise for improving precision medicine for CID patients. AI-based models can analyze large-scale multi-omics datasets that incorporate clinical, genetic, proteomic, and imaging data to refine risk prediction and facilitate personalized therapy [143,144]. Future research should focus on identifying early cardiovascular disease phenotypes in CID patients through AI-driven analysis, allowing for earlier intervention and risk reduction. In addition, the development of predictive models for cardiovascular complications using deep learning techniques could improve clinical decision making and treatment optimization. However, key challenges such as data standardization, computational resource requirements, and interpretability of AI models for clinical use must be addressed to ensure widespread adoption and clinical reliability [142].

## 7. Conclusions

CIDs significantly increase cardiovascular risk through persistent systemic inflammation, endothelial dysfunction, and immune dysregulation. Despite strong epidemiological and mechanistic evidence, current cardiovascular risk models do not fully account for chronic inflammation, highlighting the need for improved risk stratification. Early screening, lipid-lowering therapy, targeted anti-inflammatory treatments, and tailored exercise programs are essential to reduce cardiovascular events in CID patients. While biologic and targeted synthetic DMARDs show potential benefit, their long-term cardiovascular safety requires further study. The integration of AI-driven analytics and multi-omics approaches may improve risk prediction and personalized therapy. Future research should focus on refining risk assessment, evaluating novel biomarkers, and optimizing prevention strategies to improve long-term cardiovascular outcomes in CID.

## Figures and Tables

**Table 1:** Summary of clinical trials addressing cardiovascular disease risk in chronic inflammatory diseases.

Author, Year	Country	Target Disease	Sample Characteristics	Objective	Main Findings	**Comment**
Burggraaf B. et al., 2018 [58]	The Netherlands	RA	212 RA patients without CVD/diabetes, randomized to treat-to-target vs. usual care	Evaluate the impact of cardiovascular treat-to-target intervention on CIMT progression.	CIMT progression was lower in the treat-to-target group, but only in RA patients without metabolic syndrome.	Findings support targeted CVD risk reduction in RA patients without metabolic syndrome.
Burggraaf B. et al., 2019 [59]	The Netherlands	RA	320 RA patients randomized; 219 completed 5-year follow-up	Evaluate treat-to-target approach for cardiovascular risk management in RA.	Treat-to-target group had lower carotid intima–media thickness progression and fewer cardiovascular events.	Supports aggressive cardiovascular risk management in RA.
Charles-Schoeman C. et al., 2018 [60]	USA	RA	30 RA patients from the AMPLE trial randomized to abatacept or adalimumab	Assess changes in HDL proteome and function with abatacept vs. adalimumab treatment.	Both drugs improved HDL function; adalimumab increased PON1 activity and reduced HDL-associated SAA-I more than abatacept.	Highlights differential cardiovascular effects of biologic DMARDs on lipid metabolism.
Christina Charles-Schoeman et al., 2007 [61]	USA	RA	20 RA patients randomized to atorvastatin 80 mg vs. placebo for 12 weeks	Evaluate impact of atorvastatin on HDL inflammatory properties and RA disease activity.	Atorvastatin improved HDL anti-inflammatory properties but had no significant effect on RA disease activity.	Supports potential cardiovascular benefits of atorvastatin in RA despite limited impact on inflammation.
Deyab G. et al., 2021 [62]	Norway	RA	39 RA patients starting methotrexate or TNFi + methotrexate	Examine effects of DMARDs on syndecan-1, MMP-9, and TIMP-1 in RA.	Syndecan-1 levels decreased after six weeks of treatment, indicating a potential cardioprotective role.	Suggests endothelial glycocalyx-preserving effects of DMARDs may contribute to cardiovascular benefits.
Genovese M.C. et al., 2016 [63]	USA, Europe	RA	527 RA patients with inadequate response to TNFi or biologic DMARDs	Assess efficacy of baricitinib vs. placebo in refractory RA.	Baricitinib (4 mg) significantly improved ACR20 response and HAQ-DI scores; increased risk of infections and cardiovascular events.	Supports baricitinib as an option for refractory RA but highlights potential cardiovascular risks.
Giles J.T. et al., 2020 [64]	Multiple	RA	3080 RA patients with cardiovascular risk factors, followed for 3.2 years	Compare cardiovascular risk of tocilizumab vs. etanercept.	No significant difference in major cardiovascular events; increased lipid levels with tocilizumab.	Findings suggest tocilizumab does not increase cardiovascular risk despite lipid changes.
Gonzalez-Juanatey C. et al., 2006 [65]	Spain	RA	8 RA patients receiving infliximab vs. 15 RA patients on conventional therapy	Assess impact of infliximab on CIMT.	Infliximab slowed CIMT progression compared to conventional therapy.	Supports TNF inhibition for reducing cardiovascular risk in severe RA.
Haglo H. et al., 2021 [66]	Norway, USA	RA, SpA, SLE	40 patients (33 female, 7 male, mean age 48 years) with RA, SpA, or SLE	Evaluate self-administered smartphone-guided HIIT vs. supervised HIIT.	VO2max and HRQoL improved similarly in both groups, suggesting smartphone guidance is an effective alternative to supervised training.	Supports mobile app-guided HIIT as a cost-effective exercise intervention for inflammatory rheumatological disease patients.
Ikdahl E. et al., 2015 [67]	Norway	Inflammatory joint diseases (RA, AS, PsA)	85 statin-naïve patients with ultrasound-verified carotid plaques	Assess long-term effects of rosuvastatin on endothelial function and atherosclerosis.	Rosuvastatin improved endothelial function, reduced arterial stiffness, and carotid plaque height.	Supports statin therapy in inflammatory joint disease patients with established atherosclerotic disease.
Ikdahl E. et al., 2016 [68]	Norway	Inflammatory joint diseases (RA, AS, PsA)	89 patients with carotid plaques receiving rosuvastatin for 18 months	Evaluate long-term effects of rosuvastatin on arterial stiffness and blood pressure.	Rosuvastatin significantly reduced arterial stiffness and blood pressure over 18 months.	Supports intensive lipid-lowering for cardiovascular prevention in inflammatory joint diseases.
Ikonomidis I. et al., 2011 [69]	Greece	RA	46 RA patients, 23 treated with anakinra vs. 23 with prednisolone	Assess effects of anakinra on apoptotic markers and left ventricular function.	Anakinra reduced apoptotic markers, improved left ventricular performance.	Suggests IL-1 inhibition benefits myocardial function in RA.
Ikonomidis I. et al., 2019 [70]	Greece	RA	120 RA patients randomized to anakinra, tocilizumab, or prednisolone	Compare effects of IL-1 and IL-6 inhibition on myocardial and vascular function.	Anakinra improved myocardial function, while tocilizumab improved vascular function.	Findings highlight differential effects of IL-1 and IL-6 blockade in RA-associated cardiovascular risk.
Kim H.J. et al., 2015 [71]	Korea	RA	44 RA patients and 22 healthy controls, all female	Assess the impact of methotrexate CIMT.	RA patients had higher CIMT than controls; methotrexate use was associated with lower CIMT.	Supports methotrexate’s potential protective role against CVD in RA.
Kitas G.D. et al., 2019 [72]	UK	RA	3002 RA patients, mean age 61 years, 74% female	Assess whether atorvastatin reduces cardiovascular events in RA patients.	34% reduction in cardiovascular event risk with atorvastatin; significantly reduced LDL and CRP levels.	Supports atorvastatin use for primary prevention of cardiovascular events in RA.
Kristensen L.E. et al., 2023 [73]	Multiple	RA	4362 patients aged ≥50 years with RA and ≥1 cardiovascular risk factor	Identify high-risk vs. low-risk populations for tofacitinib vs. TNFi.	High risk for cardiovascular events and malignancies in older patients and smokers; no increased risk in younger non-smokers.	Findings support individualized risk assessment for tofacitinib use.
Plein S. et al., 2020 [74]	UK	RA	81 treatment-naïve RA patients randomized to etanercept + methotrexate or methotrexate alone; 30 matched controls	Assess cardiovascular impact of DMARD therapy using cardiac magnetic resonance imaging.	RA patients had impaired vascular stiffness, left ventricular mass, and myocardial fibrosis; DMARD therapy improved vascular stiffness, with no difference between treatment arms.	Supports early DMARD therapy for improving cardiovascular parameters in RA.
Rubbert-Roth A. et al., 2020 [75]	USA, Europe, Australia, Brazil	RA	612 RA patients refractory to biologic DMARDs	Compare upadacitinib vs. abatacept in RA refractory to biologic DMARDs.	Upadacitinib led to greater reductions in DAS28-CRP and higher remission rates vs. abatacept but had more adverse events.	Suggests upadacitinib as a more effective option for RA but with higher risk of serious adverse effects.
Smolen J.S. et al., 2019 [76]	Multiple (24 countries)	RA	648 patients with active RA and inadequate response to methotrexate	Assess efficacy and safety of upadacitinib monotherapy vs. methotrexate.	Upadacitinib significantly improved clinical outcomes compared to methotrexate.	Supports upadacitinib monotherapy as an option for RA.
Tam L.S. et al., 2011 [77]	Hong Kong	RA	50 RA patients randomized to rosuvastatin 10 mg or placebo for 12 months	Assess effects of rosuvastatin on carotid atherosclerosis and arterial stiffness.	Rosuvastatin improved subendocardial viability ratio but had no effect on IMT or augmentation index.	Suggests potential vascular benefits of statins in RA, but no clear effect on atherosclerosis progression.
Welsh P. et al., 2016 [78]	UK	RA	357 RA patients receiving tocilizumab or placebo	Assess IL-6 inhibition effects on NT-proBNP and hsTnT as cardiovascular biomarkers.	No significant effect of tocilizumab on NT-proBNP; hsTnT increased in treated patients.	Suggests no rapid cardiovascular biomarker benefit from IL-6 blockade despite RA disease control.
Yang M. et al., 2018 [79]	China	RA	119 active RA patients randomized to puerarin 400 mg IV or control for 24 weeks	Evaluate the effect of puerarin on CIMT and insulin resistance in RA.	Puerarin significantly reduced CIMT and improved insulin resistance without major side effects.	Suggests potential cardiovascular protective effects of puerarin in RA, but further trials needed.
Askanase A.D. et al., 2025 [80]	Multiple (22 countries)	SLE	427 SLE patients, 95% female, median age 42 years	Evaluate efficacy and safety of cenerimod in SLE.	Primary endpoint not met, but 4 mg dose showed some improvement in disease activity; well tolerated.	Further phase 3 trials are ongoing to assess efficacy in SLE.
Carlucci P.M. et al., 2018 [81]	USA	SLE	64 SLE patients and 35 healthy controls	Assess the role of SLE proinflammatory neutrophils in cardiovascular risk.	Increased vascular inflammation, arterial stiffness, and coronary plaque burden in SLE; strong association with neutrophil gene signature.	Supports role of immune dysregulation in lupus-associated cardiovascular risk.
Casey K.A. et al., 2020 [39]	USA	SLE	305 SLE patients, 99 received anifrolumab, 102 placebo	Evaluate effects of type I IFN inhibition on cardiometabolic markers.	Anifrolumab reduced neutrophil extracellular traps complexes and inflammatory markers, improved cholesterol efflux capacity.	Suggests IFN inhibition may reduce cardiovascular risk in SLE.
Fatemi et al., 2014 [82]	Iran	SLE	90 patients, randomized to atorvastatin 20 mg/day vs. placebo for 3 months	To evaluate the effect of atorvastatin on disease activity and inflammatory markers in SLE	No significant effect on disease activity, but CRP decreased and lipid profile improved in the statin group.	Cardiovascular risk markers improved, but short follow-up and no cardiovascular events reported.
Hasni S.A. et al., 2021 [83]	USA	SLE	30 SLE patients randomized to tofacitinib or placebo	Assess safety and immunological effects of tofacitinib in SLE.	Tofacitinib improved cholesterol profiles, arterial stiffness, and reduced type I IFN gene signature.	Supports further research on JAK inhibition for cardiovascular risk in SLE.
Mok et al., 2011 [84]	Hong Kong	SLE	72 SLE patients with subclinical atherosclerosis, no prior CVD	Examine the effect of rosuvastatin ± aspirin on endothelial markers and carotid atherosclerosis progression	Rosuvastatin reduced homocysteine and endothelial activation markers but had limited effect on CIMT progression over 24 months	No clinical cardiovascular endpoints; short duration and limited sample size hinder conclusions
Plazak et al., 2011 [85]	Poland	SLE	60 SLE patients, randomized to atorvastatin vs. placebo for 12 months	To evaluate atorvastatin effect on progression of coronary calcifications and myocardial perfusion.	Atorvastatin halted progression of atherosclerosis seen on multi-detector computed tomography; placebo group showed significant increase in plaque volume and calcium score.	First to demonstrate imaging-based atherosclerosis benefit of statin in SLE; small sample size but robust cardiovascular imaging endpoints.
Wallace et al., 2019 [40]	USA	SLE	298 SLE patients, autoantibody-positive, long-term follow-up up to 13 years	To assess long-term safety and efficacy of IV belimumab + standard of care in SLE patients	Long-term belimumab was well tolerated and maintained disease control. Cardiovascular deaths were reported (1 cardiac arrest, 1 coronary artery disease), but not a primary endpoint.	Focused on overall safety and SLE disease control. Cardiovascular outcomes not a primary focus but reported incidentally.
Afif et al., 2024 [86]	Multinational	UC	348 UC patients continuing subcutaneous ustekinumab for 4 years	To assess long-term efficacy and safety of ustekinumab in UC	No major adverse cardiovascular events reported through 4 years. Clinical remission and endoscopic improvement maintained.	While cardiovascular safety appears favorable, the study was not designed to assess cardiovascular outcomes specifically.
Armuzzi A. et al., 2024 [87]	Multiple (USA, Italy, etc.)	UC	796 patients with moderately to severely active UC in True North; 823 in open-label extension	Evaluate cardiovascular safety of ozanimod	No new cardiovascular safety signals, minimal changes in heart rate and blood pressure, well-tolerated cardiovascular safety profile	Supports safe use of ozanimod in UC patients per label instructions
Sandborn et al., 2018 [88]	Multi-national	Crohn’s disease	718 patients (randomized and non-randomized extension of UNITI trials)	Long-term efficacy and safety of ustekinumab	Remission maintained through 92 weeks; clinical remission ~74%, low incidence of serious adverse events and infections	No cardiovascular endpoints reported, but data support long-term inflammation control without increase in cardiovascular risk

Abbreviations: ACR, American College of Rheumatology score, measures improvement in rheumatoid arthritis, which is categorized into ACR20, ACR50, and ACR70, indicating a 20%, 50%, and 70% improvement in disease symptoms, respectively; AS, ankylosing spondylitis; CIMT, carotid intima–media thickness; CRP, C-reactive protein; CVD, cardiovascular disease; DAS28-CRP, Disease Activity Score-28 based on C-reactive protein; DMARDs, disease-modifying antirheumatic drugs; HAQ-DI, Health Assessment Questionnaire Disability Index; HDL, high-density lipoprotein; HIIT, high-intensity interval training; HRQoL, health-related quality of life; hsTnT, high-sensitivity cardiac troponin T; IFN, interferon; IL-1, interleukin-1; IL-6, interleukin-6; JAK, Janus Kinase; LDL, low-density lipoprotein; MMP-9, matrix metalloproteinase-9; TIMP-1, tissue inhibitor of metalloproteinases; NT-proBNP, N-terminal pro-brain natriuretic peptide; PON1, paraoxonase-1; PsA, psoriatic arthritis; RA, rheumatoid arthritis; SAA-I, serum amyloid A-I, SpA, spondyloarthritis; TNFi, tumor necrosis factor inhibitor; UC, ulcerative colitis; VO2max, maximal oxygen uptake.

**Table 2:** Potential strategies for risk assessment, optimization, and management in patients with chronic inflammatory diseases.

Strategy	Rationale	Potential Benefits
**Risk Assessment**		
Incorporation of inflammatory markers (CRP, IL-6, fibrinogen, PAI-1) into cardiovascular risk models	Inflammatory markers contribute to atherosclerosis and cardiovascular events.	Better prediction of cardiovascular risk in CID populations.
Use of non-invasive vascular imaging (carotid ultrasound, coronary artery calcium scoring, photon-counting computed tomography) for early detection	Early detection of vascular changes can guide preventive interventions.	Earlier intervention can reduce cardiovascular morbidity and mortality.
AI-driven risk stratification integrating clinical, genetic, and biomarker data	AI-driven models improve risk prediction by integrating multi-dimensional data.	Enhanced precision in identifying high-risk individuals.
Refinement of cardiovascular risk scores to include chronic inflammation and autoimmune diseases	Current risk scores do not fully account for the impact of chronic inflammation.	Improved accuracy in cardiovascular risk stratification.
**Optimization**		
Early screening and monitoring for subclinical atherosclerosis in CID patients	Subclinical atherosclerosis progresses even with normal LDL-c levels in CID patients.	Prevention of cardiovascular complications in seemingly low-risk patients.
Routine assessment of disease activity and systemic inflammation	Monitoring inflammation levels helps adjust treatment strategies to reduce risk.	Allows for dynamic treatment adjustments to mitigate cardiovascular risk.
Personalized medicine approach using multi-omics data (genomics, proteomics, metabolomics)	Multi-omics approaches provide personalized insights into disease progression.	Tailored interventions to reduce inflammation-driven cardiovascular events.
Regular cardiovascular follow-ups for high-risk patients, especially older individuals	Older CID patients have a cumulative risk of cardiovascular complications.	Focused monitoring reduces the likelihood of undetected cardiovascular disease.
**Management**		
Lifestyle interventions: dietary control, weight management, smoking cessation, physical activity	Lifestyle factors significantly contribute to cardiovascular risk modulation.	Long-term cardiovascular protection through lifestyle modification.
Vaccination against preventable infections (influenza, pneumococcus, COVID-19, hepatitis B, herpes zoster, HPV)	Preventable infections can trigger systemic inflammation and cardiovascular events.	Reduced infection-related inflammation and lower cardiovascular risk in CID patients.
Aggressive lipid-lowering therapy (statins with anti-inflammatory effects)	Statins have both lipid-lowering and anti-inflammatory properties.	Dual benefit of lipid reduction and inflammation control.
Hypertension and diabetes management tailored to inflammatory disease burden	Inflammation can worsen hypertension and diabetes outcomes, requiring tailored management.	Optimized control of metabolic comorbidities improves overall prognosis.
Microbiome modulation strategies (probiotics, dietary interventions, gut-targeted therapies)	Gut microbiota plays a role in systemic inflammation and metabolic health.	Potential reduction in systemic inflammation through gut-targeted therapies.
Use of SGLT2 inhibitors and GLP-1 receptor agonists for metabolic and cardiovascular protection if appropriate	These agents provide cardiometabolic benefits, including improved glucose control, weight loss, and vascular health.	Reduction in cardiovascular events and improved metabolic profile in CID patients with diabetes or obesity.
Stress management and psychological support	Chronic stress and depression contribute to systemic inflammation and cardiovascular risk.	Improved cardiovascular and psychological well-being through stress reduction technique.
Dietary sodium reduction and increased potassium intake	Excess sodium intake exacerbates hypertension and cardiovascular risk.	Lowered blood pressure and reduced cardiovascular disease burden.
Routine anemia screening and iron management	Anemia is common in CID and is associated with increased cardiovascular risk.	Improved oxygen delivery and reduced cardiovascular strain.

Abbreviations: AI, artificial intelligence; CID, chronic inflammatory disease; CRP, C-reactive protein; IL-6, interleukin-6; LDL-c, low-density lipoprotein cholesterol; PAI-1, plasminogen activator inhibitor-1.

**Table 3:** Key research priorities in cardiovascular risk and chronic inflammatory diseases.

Research Area	Key Questions	Potential Impact
Risk prediction models	How can inflammatory biomarkers, PRS, and multi-omics improve cardiovascular risk assessment?	Personalized, CID-specific cardiovascular risk scores
Primary vs. secondary prevention	Does chronic inflammation drive first-time CVD events as aggressively as recurrent events?	Refined preventive treatment thresholds
Anti-inflammatory therapies	What are the long-term cardiovascular effects of TNF, IL-6, and IL-1 blockers?	Optimized therapeutic strategies for CID patients
Gut microbiome and CVD	Can microbiota-targeted interventions reduce systemic inflammation and atherosclerosis?	Novel dietary and therapeutic interventions
Advanced imaging	How can PCCT and CAC scoring be integrated into CID cardiovascular risk models?	Improved early detection and intervention
Artificial Intelligence	Can AI predict cardiovascular complications in CID patients?	Precision medicine and real-time risk assessment

Abbreviations: AI, artificial intelligence; CAC, coronary artery calcium; CID, chronic inflammatory disease; CVD, cardiovascular disease; IL-1, interleukin-1; IL-6, interleukin-6; PCCT, photon-counting computed tomography; PRS, polygenic risk score; TNF, tumor necrosis factor.
